# Effects of Ligands in Rare Earth Complex on Properties, Functions, and Intelligent Behaviors of Polyurea–Urethane Composites

**DOI:** 10.3390/polym14102098

**Published:** 2022-05-21

**Authors:** Lu Zhou, Hongwei Yang, Zhen Zhang, Yue Liu, Jayantha Epaarachchi, Zhenggang Fang, Liang Fang, Chunhua Lu, Zhongzi Xu

**Affiliations:** 1 College of Materials Science and Engineering, Nanjing Tech University, Nanjing 211816, China; 201961103003@njtech.edu.cn (L.Z.); 201961203115@njtech.edu.cn (H.Y.); a773580419@163.com (Z.Z.); lfang@njtech.edu.cn (L.F.); chhlu@njtech.edu.cn (C.L.); 2 State Key Laboratory of Materials-Oriented Chemical Engineering, Nanjing Tech University, Nanjing 211816, China; 3 Jiangsu Collaborative Innovation Center for Advanced Inorganic Function Composites, Nanjing Tech University, Nanjing 211816, China; 4 Institute of Active Polymers, Helmholtz-Zentrum Hereon, Kantstr. 55, 14513 Teltow, Germany; yue.liu@hereon.de; 5 School of Engineering, University of Southern Queensland, Toowoomba, QLD 4350, Australia; jayantha.epaarachchi@usq.edu.au

**Keywords:** rare earth organic complexes, ligands, dispersion, mechanical property, fluorescence intensity

## Abstract

There is a need to create next-generation polymer composites having high property, unique function, and intelligent behaviors, such as shape memory effect (SME) and self-healing (SH) capability. Rare earth complexes can provide luminescence for polymers, and their dispersion is highly affected by ligand structures. Here, we created three different REOCs with different ligands before studying the effects of ligands on REOC dispersion in polyurea–urethane (PUU) with disulfide bonds in main chains. In addition, the effects of different REOCs on mechanical properties, luminescent functions, and intelligent behaviors of PUU composites were studied. The results showed that REOC I (Sm(TTA)_3_phen: TTA, thenoyltrifluoroacetone; phen, 1,10-phenanthroline) has incompatible ligands with the PUU matrix. REOC I and REOC III (Sm(BUBA)_3_phen: BUBA, 4-benzylurea-benzoic acid) with amine and urea groups facilitate their dispersion. It was REOC III that helped the maintenance of mechanical properties of PUU composites due to the good dispersion and the needle-like morphologies. Due to more organic ligands of REOC III, the fluorescence intensity of composite materials is reduced. The shape recovery ratio of the composite was not as good as that of pure PUU when a large amount of fillers was added. Besides, REOC I reduced the self-healing efficiency of PUU composites due to poor dispersion, and the other two REOCs increased the self-healing efficiency. The results showed that ligands in REOCs are important for their dispersion in the PUU matrix. The poor dispersion of REOC I is unbeneficial for mechanical properties and intelligent behavior. The high miscibility of REOC II (Sm(PABA)_3_phen: PABA, 4-aminobenzoic acid) decreases mechanical properties as well but ensures the good shape recovery ratio and self-healing efficiency. The mediate miscibility and needle-like morphology of REOC III are good for mechanical properties. The shape recovery ratio, however, was decreased.

## 1. Introduction

Shape memory (SM) and self-healing (SH) polymers are two widely reported intelligent polymeric materials in the last decades [1,2]. To date, different polyurethanes (PUs) with various chemical structures are reported, which present both shape memory effect (SME) and SH behaviors due to the choice of diols (polyols), isocyanates, and chain extenders [3,4,5]. The incorporation of such intelligence into PUs enriches their application fields and prolongs service life.

Properties, especially mechanical properties, must be a concern when intelligent polymers are used. Unfortunately, shape recovery of shape memory polymers (SMPs) usually occurs upon heating, while low force output is generated and applications are accordingly restricted. Besides, structural requirement for SH is against high mechanical properties because high chain mobility facilitates good recovery of scratches or cracks. The reinforcement of mechanical properties, thus, is important [6,7,8]. As far as PUs are concerned, carbon fiber [9], graphene [10], carbon nanotubes [11], clay [12], silica [13], nanocellulose [14], and so forth are blended to increase their strength and modulus. Similar fillers are used as well for SH polyurethanes [15,16].

In addition to intelligence and property, function is another important research topic in polymeric materials, while fillers are usually required as well. After the incorporation of carbon-based or other inorganic fillers, thermal conductivity of polymer composites can be increased [17,18]. Carbon-based fillers or metallic powders can improve electric conductivity or provide EMI shielding capability [19,20,21,22]. In addition, a rare earth organic complex (REOC) is compounded with polymers to prepare rare-earth-based polymer composites with luminescent functions [23,24,25]. Such composites not only have the unique excellent luminescent characteristics of REOC, but also present the features of polymers, including easy processing and shape deformation. Composite materials have been widely used in the fields of light conversion films for agriculture and solar cells, anticounterfeiting mark, biomedicine, light-emitting device, and so on [26,27,28].

The mixing of REOC with intelligent PUs can bring luminescent functions. The dispersion of REOC in PUs must be a concern to ensure the maintenance of mechanical properties. To date, different approaches have been used to facilitate the dispersion of REOC in the polymer matrix [29,30]. Due to high crystallinity, REOC is hard to be mixed with the polymer matrix in a physical manner. Poor compatibility usually decreases the mechanical properties of polymer composites. The ligands on REOC can be optimized to have reactive functional groups, usually double bonds, to achieve copolymerization with polymers in a chemical manner [31]. The dispersion of REOC in polymers can be improved. However, due to large steric hindrance of REOC, a large amount of loading is limited. In addition, ligands are introduced into polymeric main chains and form coordination bonds with rare earth ions [25]. The dispersion can be further improved, while the original chemical structures of polymers are changed. More recently, our group created a new REOC with ligands that can form hydrogen bonds with polymers, and the mechanical properties can be highly increased [32].

In this manuscript, three different REOCs are used as luminescent fillers for intelligent PU materials with SME and SH behavior. The effects of ligands in the dispersion of REOCs in PU materials having disulfide bonds were investigated. In addition, the mechanical properties, luminescent properties, shape memory capabilities, and self-healing behaviors of the composites with varied loading contents were characterized.

## 2. Experimental Section

### 2.1. Materials

Samarium nitrate hexahydrate, thenoyltrifluoroacetone (HTTA), sodium hydroxide, dibutyltin dilaurate (DBTDL), and methanol were purchased from Sinopharm Chemical Reagent Co., Ltd., Beijing, China. 4-Aminophenyl disulfide (AFD), benzyl isocyanate, and 4-aminobenzoic acid (PABA) were obtained from Shanghai Aladdin Biochemical Technology Co., Ltd., Shanghai, China. Isophorone diisocyanate (IPDI) and N,N-dimethylformamide (DMF) were acquired from Xilong Chemical Reagent Co., Ltd., Guangzhou, China. 1,10-Phenanthroline (Phen) and polycarbonate diol (PCDL, M_n_ = 1000 Da) were purchased from Jinan Hua Kai Resin Co., Ltd., Jinan, China. Ethanol was obtained from Shanghai Hongtu Chemical Reagent Factory.

### 2.2. Preparation of Polyurea–Urethane (PUU)

**Scheme 1:**
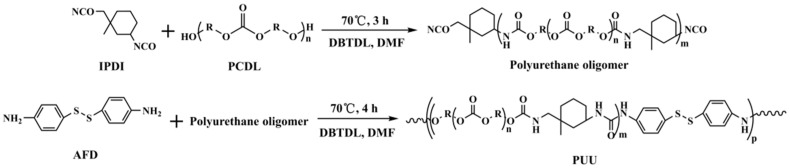
Synthesis procedures of PUU.

The preparation of PUU has been reported in a previous work [33]. Amounts of 14.466 g (0.01 mol) of PCDL, 7.235 g (0.03 mol) of IPDI, 2–3 drops of DBTDL catalyst, and 15 mL DMF were added into a three-necked flask, and the reaction was carried out in a water bath of 70 °C for 3 h. Then, 0.449 g (0.002 mol) of AFD, 2–3 drops of DBTDL catalyst, and 15 mL of DMF were added into the three-necked flask to undergo further reaction for another 4 h. Finally, the resulting solution was poured into a PTFE plate and put in an oven at 60 °C for 48 h. In Scheme 1, the synthesis procedure of PUU is shown.

### 2.3. Preparation of REOC I: Sm(TTA)_3_(Phen)

According to a molar ratio of 1:3:1, 8.888 g (0.02 mol) samarium nitrate hexahydrate, 13.3332 g (0.06 mol) HTTA, 3.9644 g (0.02 mol) Phen, and 4 g (0.10 mol) sodium hydroxide were dissolved in 60, 300, 60 and 100 mL ethanol, respectively. First, the solutions of HTTA and samarium nitrate hexahydrate were put into a three-necked flask and reacted for 1 h in a water bath of 60 °C. Then, the Phen solution was added to continue the reaction for 30 min. Sodium hydroxide solution was added dropwise to adjust the pH value to 6–7, and the reaction continued for 4.5 h. Finally, the product was centrifuged and washed with water and ethanol three times, respectively. The products were placed in a vacuum oven at 60 °C for 48 h.

### 2.4. Preparation of REOC II: Sm(PABA)_3_(Phen)

According to a molar ratio of 1:3:1, 4.444 g (0.01 mol) samarium nitrate hexahydrate, 4.1142 g (0.03 mol) PABA, and 1.9822 g (0.01 mol) Phen were separately dissolved in 20 mL methanol each. An amount of 2 g (0.05 mol) of sodium hydroxide was dissolved in 100 mL deionized water. First, samarium nitrate hexahydrate solution was added into a three-necked flask and placed in a 60 °C water bath. Then PABA solution was mixed with Phen solution, followed with the addition of sodium hydroxide solution, to make a pH value between 5 and 6. The product of the reaction after 1 h was poured into the PTFE lining and placed in a hydrothermal oven for 4 h at 120 °C. Finally, the solid was filtered and washed with methanol for 2–3 times. The powder was dried in a vacuum oven at 60 °C for 48 h.

### 2.5. Preparation of REOC III: Sm(BUBA)_3_(Phen)

According to a molar ratio of 1:2, 1.5767 g (0.01 mol) benzyl isocyanate and 1.5 g (0.02 mol) of prepared REOC II were added into a sample bottle, followed by the addition of 15 mL DMF. The whole mixed solution was placed in a 60 °C water bath and reacted for 6 h. Then a certain amount of deionized water and the solution after complete reaction was poured into a beaker, where a large amount of white precipitate appeared after a while. After centrifugation, the powder was finally put in a vacuum oven at 60 °C and dried for 48 h.

### 2.6. Preparation of REOC/Polyurethane Composites

An amount of 5 g (0.003 mol) of PUU (M_w_ = 111.5 kDa, PDI = 1.87) was dissolved in DMF and put in an oven at 60 °C until completely dissolved. The contents of three kinds of REOCs were set as 0, 2, 5, 10, and 20 phr (per hundred resin). REOC was added to the polyurethane solution and mixed under magnetic stirring for a period of time. Finally, these mixed solutions were poured into Petri dishes with the same size. The composite material was obtained after water evaporation and sufficiently dried in a vacuum oven.

### 2.7. Characterizations

A Fourier-transform infrared spectrometer (FTIR, Vector 22, Bruker, Billerica, MA, USA) was used to examine the chemical structure of REOC. The sample was mixed with potassium bromide (KBr) and then pressed into a test piece. The chemical structure of REOC/PUU composites was determined by attenuated total reflection (ATR) attachment of FTIR.

The thermal stability of REOC and REOC/PUU composites was characterized by a thermogravimetric analyzer (TGA, Netzsch STA 449C, Thermo, Tokyo, Japan). The samples were scanned from room temperature to 800 °C in the air flow at a heating rate of 10 °C min^−1^.

A fluorescence spectrophotometer (Fluorog 3, Tokyo, Japan) was applied to measure the fluorescence intensity and lifetime of REOC and REOC/PUU composites with an excitation light of 340 nm wavelength.

The morphology of REOC and REOC/PUU composites was measured using a JSM-5900 scanning electron microscope (SEM). The dispersion of Sm^3+^ and C elements in the REOC/PUU composites with a content of 20 phr REOC was characterized.

A tensile testing machine (MZ-2000c of Jiangdu Mingzhu, Yangzhou, China) was used to evaluate the mechanical properties of REOC/PUU composites at a tensile speed of 20 mm min^−1^. Three dumbbell-shaped samples were used for each material, and the average values were analyzed.

A dynamic mechanical analyzer (DMA, MCR302, Anton Paar, Shanghai, China) was used to evaluate the *T*_g_ of the sample with 1 Hz and 0.1% vibration amplitude. The static content was set to 1 N, and the measurement was measured from −75 to 150 °C with a heating rate of 3 °C min^−1^.

The crystallization of REOC and REOC/PUU composites was observed with a radiation X-ray diffraction (XRD) instrument (Smartlab TM 9 kW, Akishima-shi, Tokyo, Japan). The scanning angle of 2θ was between 5° and 60°, and the scanning speed was 10° min^−1^.

The shape memory behavior was tested, and the sample was first clipped in a self-made mold and heated to 120 °C for 7 min until the strain was 100%. After the sample was completely cooled, its fixed length was measured. After that, the sample was put in an oven and heated from 30 to 120 °C. After waiting for 10 min at intervals of 10 °C, the sample recovery was measured and its shape recovery rate was calculated.

Finally, the self-healing performance of the sample was tested. An incision of 20–40% of the thickness was cut on the sample with a scalpel. Then the samples were heated for 5 h on a heating plate at 130 °C, and the repairing effect was verified by tensile test.

## 3. Results and Discussion

**Scheme 2:**
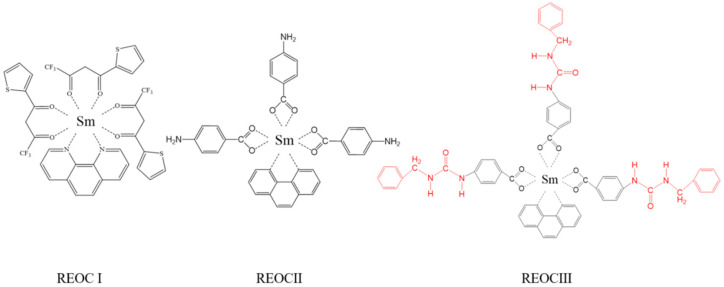
Chemical structure of REOC I (Sm(TTA)_3_phen), REOC II (Sm(PABA)_3_phen), and REOC III (Sm(BUBA)_3_phen).

Three different REOCs with varied ligands were prepared and mixed with PUU having disulfide bonds in the main chains. As shown in Scheme 2, REOC I has neglected interaction with PUU, and the ending amine groups in REOC II and urea groups in REOC III are anticipated to facilitate the dispersion of REOC in PUU. The corresponding composites are named as PUU-I, PUU-II, and PUU-III. Different loading contents of 2–20 phr were used, and the PUU composite samples are named as PUU-x-y (x: I, II, III; y: 2, 5, 10, 20).

### 3.1. Chemical Structure and Properties of Three REOCs

**Figure 1:**
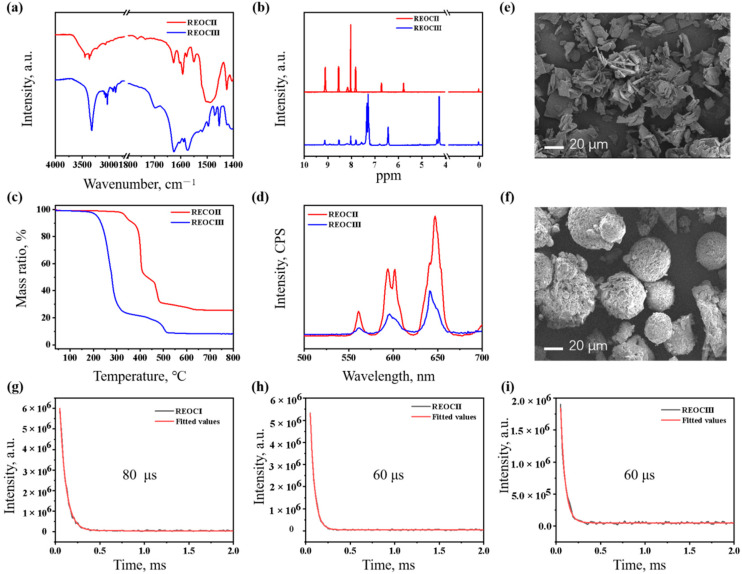
(**a**–**c**) FTIR spectra: (**a**) HNMR (**b**) and TGA (**c**) curves of REOC II and REOC III; (**d**) emission spectra of REOC II and REOC III under 340 nm UV light; (**e**,**f**) SEM images of (**e**) REOC II and (**f**) REOC III; (**g**–**i**) luminescent lifetime and fitting curves of (**g**) REOC I, (**h**) REOC II, and (**i**) REOC III.

The chemical structure of REOC I has been reported in a previous paper [34]. The full FTIR spectra are shown in Supporting Information Figure S1. The FTIR spectra at 4000–1400 cm^−^^1^ of REOC II and REOC III are shown in Figure 1a. The two characteristic peaks at 3365 and 3462 cm^−1^ correspond to the amino groups in PABA ligand in REOC [35]. On the other hand, after the reaction of REOC II with benzyl isocyanate, the tensile vibration peak of N–H in the urea group appears at 3320 cm^−1^ in REOC III [36], and a new peak at 1700 cm^−1^ indicates the C=O bond in the urea group [37]. In addition, the symmetric vibration peak of the C–H bond in the methylene group is 2873 cm^−1^, and the asymmetric vibration peak is 2920 cm^−1^ [38]. The peaks at 3031 and 3062 cm^−1^ are generated by the vibration mode of C–H in an aromatic ring [39], and their intensity increases with the further introduction of the aromatic ring in REOC III. The 1H-NMR spectra of REOC II and REOC III are presented in Figure 1b. High-resolution NMR spectra are shown in Supporting Information Figures S2 and S3. A peak between 7.5 and 8.5 ppm is related to the Phen ligands in REOC II and REOC III. The aromatic ring in REOC II and REOC III is 6.72 and 6.44 ppm, respectively [40]. After the reaction, a 5.77 ppm peak of the amino group in REOC II disappears [41], and a new peak of the methylene group is located at 4.26 ppm.

The thermal stability of REOC I has also been reported in a previous paper. The TGA curve shows two main thermal decomposition steps of REOC II and REOC III (Figure 1c). The thermal stability of REOC III with a long ligand is lower than that of REOC II with a short ligand of PABA. In addition, 25.6 wt% residues are found in REOC II, which are mainly Sm_2_O_3_ and higher than the theoretical value of 23.4%. In the case of REOC III, theoretically, 15.2 wt% of Sm_2_O_3_ should be retained after decomposition; however, only a residue of 8.3 wt% is found in the measurement. This result may be caused by a reaction between benzyl isocyanate and uncoordinated PABA, which may result in the production of additional organic products in REOC III.

Figure 1d shows the emission spectra of REOC II and REOC III under 340 nm UV light. It can be seen that the fluorescence intensity of REOC III is lower than that of REOC II, which is mainly due to the decrease in fluorescence intensity caused by long ligands of REOC III. A previous paper also has reported that REOC I has a sheet structure with a size of about 15 μm. As can be seen from the SEM images of REOC II and REOC III (Figure 1e,f), REOC II with a PABA ligand has a sheet structure similar to REOC I. However, relatively long ligands may promote aggregation, resulting in the morphology of REOC III becoming a spherical shape with a diameter of 20–40 μm.

The fluorescence lifetime decay curves of the three REOCs are shown in Figure 1g–i. The lifetime can be calculated from this curve using Equation (1), where *I* is the fluorescence intensity of time *t* and *τ* is the lifetime. The lifetime of REOC I, REOC II, and REOC III is analyzed as 80, 60 and 60 μs, respectively.

(1) $$I=I_{0}\exp\left(-t/\tau \right)$$

### 3.2. Dispersion of REOC in the PUU Matrix

**Figure 2:**
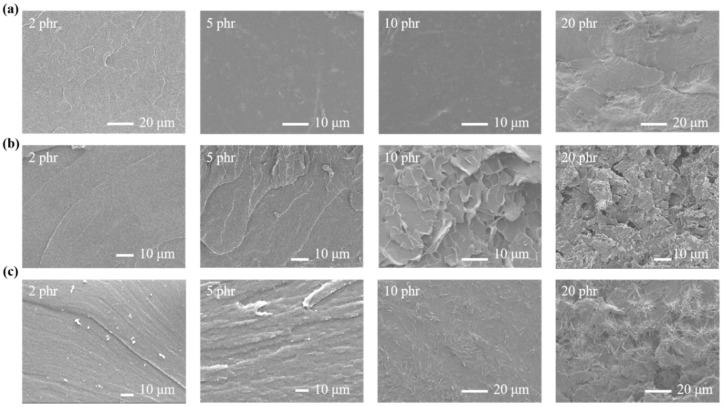
SEM images of REOC/PUU composites: (**a**) REOC I, (**b**) REOC II, (**c**) REOC III (2, 5, 10, 20 phr).

PUU composite materials were prepared by solution mixing method, and four different contents (2, 5, 10, and 20 phr) of REOC and PUU were mixed, respectively. The composite material can be obtained after drying at 60 °C. The morphologies of REOC/PUU composites were characterized by SEM, as shown in Figure 2. It can be seen that the composite PUU-I-2 shows a trace amount of REOC particles on the fracture surface, while the further increase in REOC content leads to an obvious existence of REOC particles in PUU-I-5 and PUU-I-10. A rough fracture surface is found in PUU-I-20. Neglected REOC II particles are found in PUU-II-2–PUU-II-10. Some small REOC II particles are found on the rough fracture surface of PUU-II-20. As mentioned above, the morphology of REOC III is a spherical shape with a diameter of 20–40 μm. Fewer REOC III particles can be found on the fracture surfaces of PUU-III-2 and PUU-III-5. Interestingly, needle-like particles appear in PUU-III-10 and PUU-III-20 samples.

**Figure 3:**
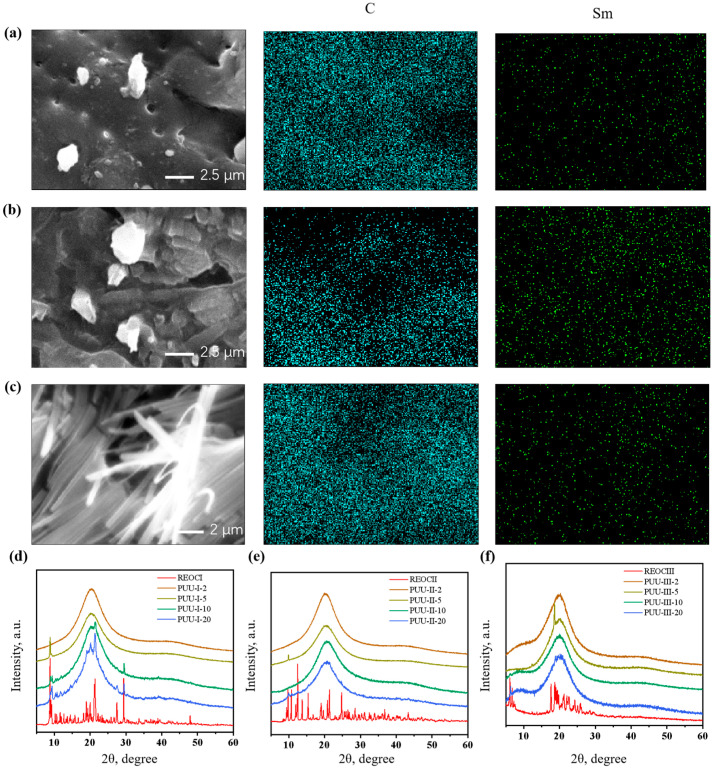
SEM and EDS images (C and Sm) of 20 phr REOC/PUU composites (**a**) REOC I, (**b**) REOC II, (**c**) REOC III; (**d**–**f**) XRD patterns of REOC/PUU composite materials (**d**) REOC I, (**e**) REOC II, (**f**) REOC III.

In order to further determine the dispersion of REOC in the PUU matrix, PUU-I-20, PUU-II-20, and PUU-III-20 are tested by scanning electron microscopy spectroscopy analysis (EDS), and the results are shown in Figure 3a–c. Although obvious particles can be found on the fracture surface of SEM, the element distribution is still uniform. The XRD patterns of three kinds of REOCs and their composites are shown in Figure 3d–f. REOC II and REOC III also present crystal structures similar to those of REOC I. An obvious peak is found in PUU-I-5–PUU-I-20, which indicates that REOC I crystals appear in these three kinds of composites. In addition, only PUU-II-20 has obvious peaks at 2θ = 9.96°, 10.75°, 12.48°, 21.38°, and 24.71° (Figure 3e). It is hard to determine the assignments of these peaks due to the complicated crystalline structures of REOC. REOC II tends to form crystals only at a high amount of loading, suggesting their high compatibility with PUU. As shown in Figure 3f, although some REOC particles appear, many obvious crystallization peaks are not found in the XRD pattern of PUU-III composites. New crystalline structures may form after their precipitation from PUU solution. The change in loading content may cause evolution of crystalline structures of REOC III, causing an evident peak in PUU-III-5, which disappeared in other samples.

**Scheme 3:**
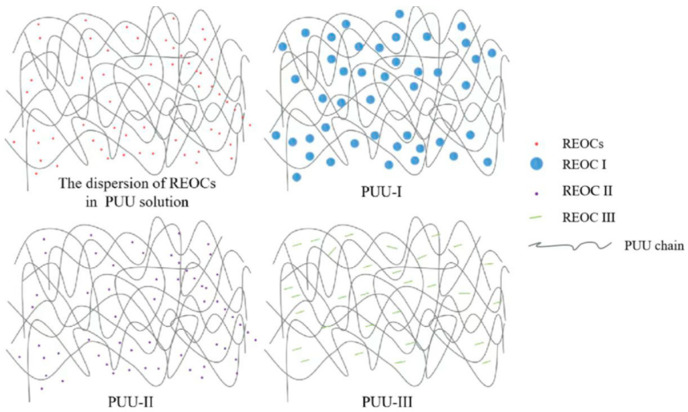
The dispersion of REOC I (Sm(TTA)_3_phen), REOC II (Sm(PABA)_3_phen), and REOC III (Sm(BUBA)_3_phen) in PUU solution and the PUU matrix.

According to the results of SEM, EDS, and XRD, the morphological evolution of three REOCs in the PUU matrix can be inferred. These ligands play a key role in determining the solubility and dispersion form of REOC in the polymer matrix. TTA and Phen are used as ligands in REOC I, both of which are incompatible with PUU. Therefore, at a low content of 5 phr, REOC I begins to separate from the PUU matrix. Meanwhile, REOC particles appear in SEM images, and crystal peaks are found in XRD patterns. The amino group from PABA acts as a ligand to facilitate the dissolution of REOC II in PUU. Furthermore, REOC II has not been clearly found in composites with a high content of 20 phr. The urea group in the ligand improves the solubility of REOC III. However, long ligands may reduce compatibility. The uniform distribution of Sm elements in EDS test results shows that the re-emergence of REOC on the fracture surface is similar to precipitation from supersaturated solution. Besides, REOC III changed to needle-like morphologies after the separation. More specifically, REOC III that precipitated from preparation solvent tends to form a spherical particle, as shown in Figure 1f. When the same compound was soluble in PUU solution, the change in surrounding environments and the large viscosity of polymers enabled REOC III to form needle-like morphologies after the precipitation. The evolutions of different REOCs in PUU solution and the PUU matrix are shown in Scheme 3.

### 3.3. Properties of PUU Composites with Three Kinds of REOC

**Figure 4:**
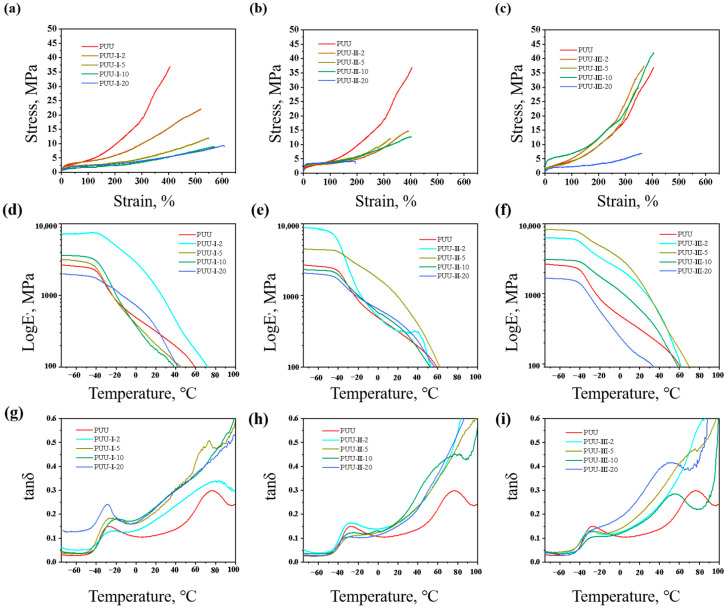
Stress–strain curves of PUU and REOC/PUU composites: (**a**) REOC I, (**b**) REOC II, (**c**) REOC III (2, 5, 10, 20 phr); (**d**–**f**) storage modulus of REOC/PUU composites with (**d**) REOC I, (**e**) REOC II, and (**f**) REOC III (2, 5, 10, 20 phr); (**g**–**i**) loss factor of PUU and REOC/PUU composites with (**g**) REOC I, (**h**) REOC II, and (**i**) REOC III (2, 5, 10, 20 phr).

Figure 4a–c presents the stress–strain curves of PUU and REOC/PUU composites. The tensile strength of pure PUU is 42 MPa, and the elongation at break is 405%. When 2–20 phr REOC I was used, the tensile strength gradually decreases from 21 to 8 MPa accordingly (Figure 4a), indicating that REOC I greatly reduces the mechanical properties of PUU-I composites. Interestingly, the mechanical properties of PUU-II also decrease with the increase in REOC II content, as the tensile strengths of PUU-II-2 and PUU-II-5 are only 15 and 13 MPa (Figure 4b). In contrast, the mechanical properties of PUU-III are higher than those of pure PUU, except for 20 phr REOC III. The tensile strengths of PUU-III-2, PUU-III-5, and PUU-III-10 are 38, 30, and 41 MPa, respectively (Figure 4c). The corresponding mechanical properties are listed in Table 1.

Then, the dynamic mechanical properties of the composites with three kinds of REOC were studied. As shown in Figure 4d–f, after the addition of a small amount of REOC, the storage moduli (E′) of PUU composites are improved to some extent. However, for composites with high REOC content, the corresponding E′ decreases. In general, compared with pure PUU, the storage moduli of the composites are improved moderately after adding REOC. In addition, as shown in Figure 4g–i, PUU has an obvious *T*_g_ at −27 °C and a wide *T*_g_ at 43–94 °C, with a peak value at around 75 °C.

After adding a small amount of REOC I, we find that the PUU-I-2 sample shows a broad *T*_g_ peak at −42 to −13 °C, with peaks at around −28 °C and around 76 °C. Interestingly, when 5, 10, and 20 phr of REOC I are added, the *T*_g_ of the original high-temperature zone disappears, and we speculate that it might be because REOC I has good compatibility with polyurethane. When adding REOC II, we can also see that due to the good compatibility between REOC II and PUU, there is no *T*_g_ in the high-temperature region of PUU-II composites. Besides, the *T*_g_ of the PUU-II-10 sample in the high-temperature region also shifts to the left, and the peak value is around 61 °C. In addition, compared with pure PUU, these samples have a broader *T*_g_ at low temperature. When adding REOC III, we find that the composites of PUU-III with 2 and 5 phr of REOC III still have no *T*_g_ at high temperature, while when 10 and 20 phr of REOC III are added, the corresponding wider *T*_g_ appears at 17–76 °C and 8–70 °C, respectively. It can be seen that with the increase in the number of parts, the *T*_g_ in the high-temperature region shifts to the left. It is speculated that the addition of excess REOC III acts as a plasticizer to a certain extent so that the entire material forms a *T*_g_ in the low-temperature zone.

**Figure 5:**
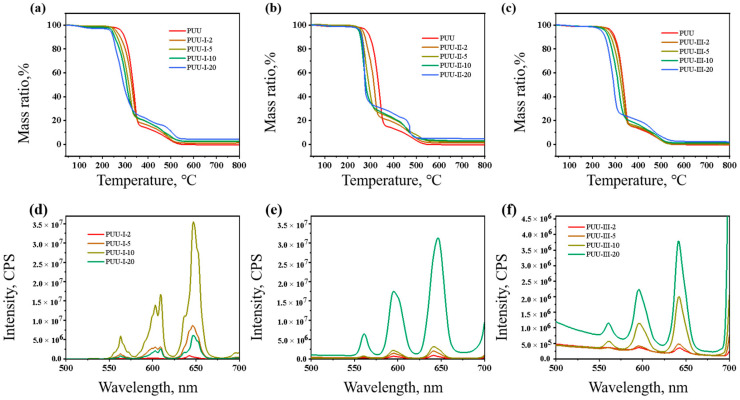
TGA (thermogravimetric analysis) curves of PUU and REOC/PUU composites with (**a**) REOC I, (**b**) REOC II, (**c**) REOC III (2, 5, 10, 20 phr); (**d**–**f**) fluorescence function spectra of REOC/PUU composites with (**d**) REOC I, (**e**) REOC II, and (**f**) REOC III (2, 5, 10, 20 phr) under 340 nm ultraviolet light.

The stabilities of the composite materials were examined by TGA, and the results are shown in Figure 5a–c. It can be seen that pure PUU decomposed at nearly 300 °C. After adding the three REOCs to the PUU, it is clear that these composites decompose in advance compared with pure PUU. The three pristine REOCs have different thermal decomposition starting temperatures (REOC I: 263 °C; REOC II: 350 °C; REOC III: 250 °C). The similar decomposition temperatures of PUU composites might be related to the rare earth ions that prompt the decomposition of polymer chains. The results show that REOC III promotes the thermal stability of the composite, which may relate to the long ligand of REOC III that may delay the thermal decomposition of PUU-III composites.

### 3.4. Functions of PUU Composite with Three Kinds of REOC

When three kinds of synthesized REOC were added into PUU, their fluorescence function also changed obviously. The fluorescence spectra of these composite materials are shown in Figure 5d–f. Under the excitation of 340 nm ultraviolet light, three obvious emission peaks belonging to Sm^3+^ are found at 561, 595, and 646 nm, respectively. With the increase in Sm^3+^ content, the fluorescence intensities of composite materials with different Sm^3+^ contents increase continuously. We can find that the fluorescence intensity of the PUU-III composite is lower than that of the PUU-II composite. It may be considered that it is due to an increase in ligands, while the number of central ions is unchanged, so that its luminescent intensity is correspondingly weakened. In other words, the absorption of UV light and the transfer to central Sm ions are limited using BUBA as ligands.

Besides comparing the fluorescence intensity of the composite materials, the fluorescence lifetime of each composite material is also analyzed (Supporting Information Figures S4–S6). At the same time, the lives of three kinds of REOC composites are calculated by Equation (1) and listed in Table 1.

It is found that when 2 phr REOC is added into PUU, the lives of the three composites are shorter than that of pure REOC. It is speculated that the addition and the good dispersion of little REOC powder in the composites can contribute to the electronic transition. With the increase in REOC content, PUU-I-5 and PUU-I-10 show the same life as pure REOC I, but the life of PUU-I-20 is somehow shortened. PUU-II-20 and PUU-III-20 have the same life span as pure REOC at 20 phr, which may be due to the agglomeration of REOC II and REOC III in PUU.

### 3.5. Intelligence of Polyurea–Polyurethane Composites with Three Kinds of REOC

**Figure 6:**
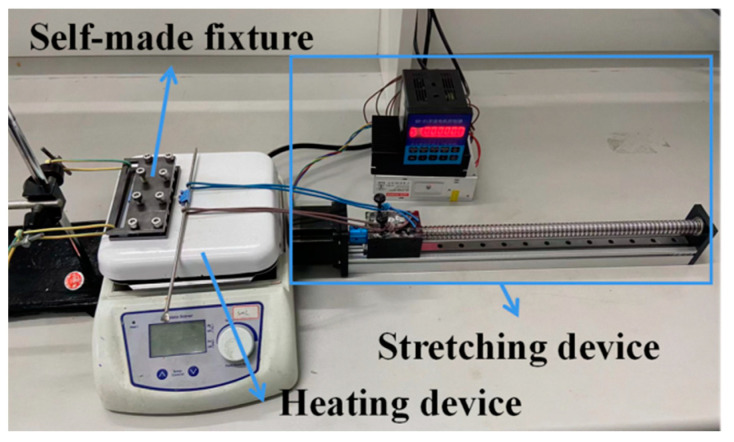
A self-made equipment for testing shape memory.

The shape memory effects of PUU and other samples are tested by a self-made equipment (Figure 6), and the shape memory effect of each sample is tested by shape recovery rate (Table 1). It is calculated as Equation (2).

(2) $$R_{r}=\frac{L_{m}-L_{p}}{L_{m}-L_{0}}\times 100\%$$

**Figure 7:**
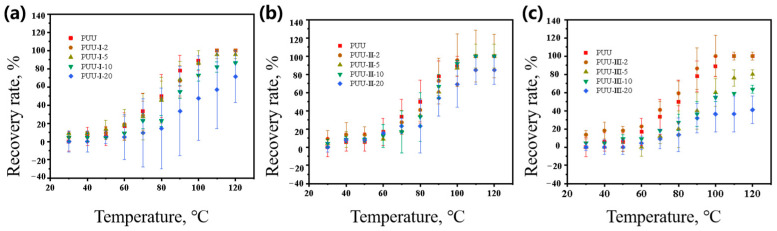
Shape recovery rates of PUU and REOC/PUU composites at different temperatures: (**a**) REOC I, (**b**) REOC II, (**c**) REOC III (2, 5, 10, 20 phr).

*L*_p_ is the length of shape recovery after heating, and *L*_0_ is the initial length of the sample as 25 mm. *L*_m_ is obtained by heating the sample at 120 °C for 7 min and stretching the sample to the strain of 100%. Shape recovery rates of PUU and REOC/PUU composites at different temperatures are shown in Figure 7. We can see that the shape recovery rate (*R*_r_) of PUU after heating to 120 ℃ is close to 100%. However, with the addition of three kinds of REOC, the *R*_r_ of composite materials obviously decreases as the number of REOCs increases. Especially for PUU-III-10 and PUU-III-20, whose *R*_r_ is about 60% and 40%, respectively, the recovery performance obviously decays. It is speculated that REOC III acts as a plasticizer in composite materials, which affects the shape recovery effect of samples. However, the *R*_r_ of composites with a small amount of REOC I and REOC II is about 80%.

**Figure 8:**
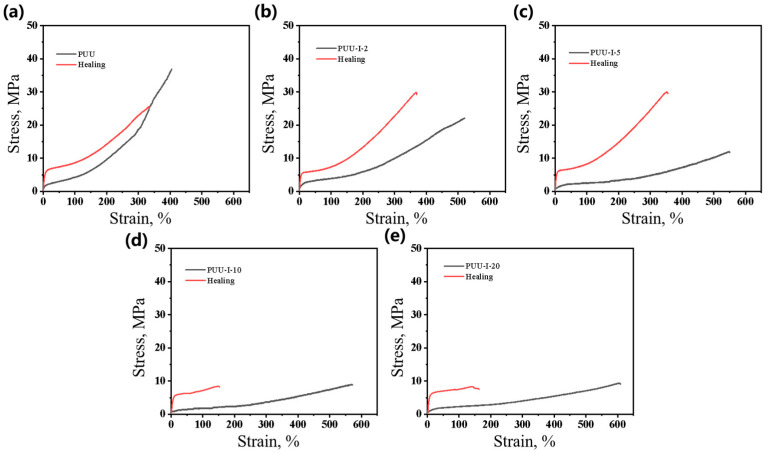
Stress–strain curves of PUU and PUU-I composite materials before and after heating and repairing at 130 °C: (**a**) PUU, (**b**) PUU-I-2, (**c**) PUU-I-5, (**d**) PUU-I-10, (**e**) PUU-I-20.

**Figure 9:**
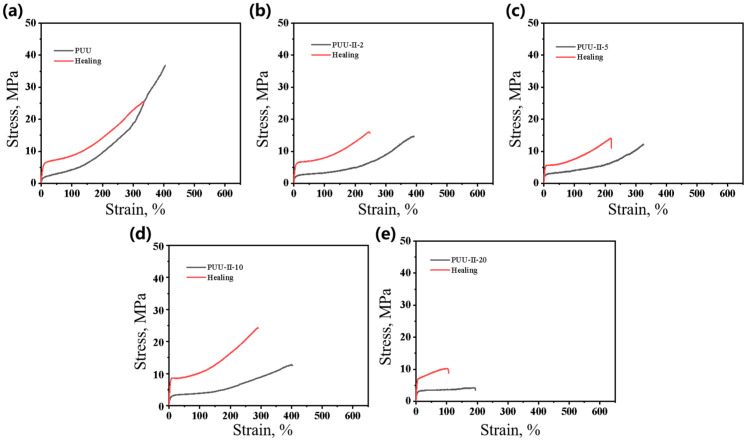
Stress–strain curves of PUU and PUU-II composite materials before and after repairing at 130 °C: (**a**) PUU, (**b**) PUU-II-2, (**c**) PUU-II-5, (**d**) PUU-II-10, (**e**) PUU-II-20.

**Figure 10:**
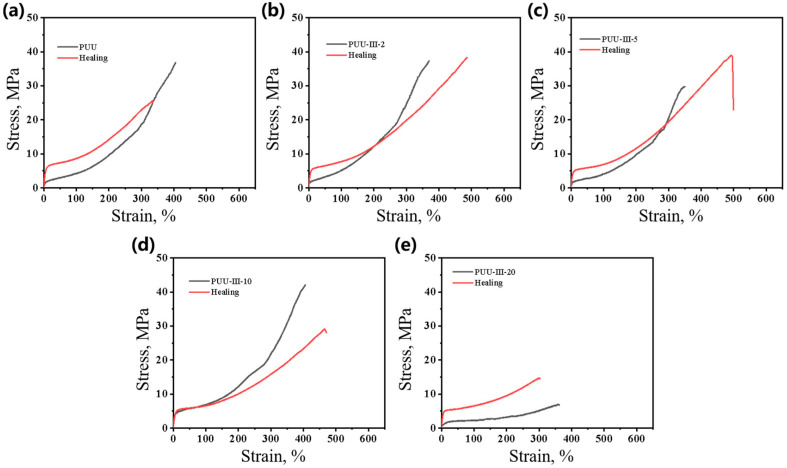
Stress-strain curves of PUU and PUU-III composite materials before and after repairing at 130 °C (**a**) PUU, (**b**) PUU-III-2, (**c**) PUU-III-5, (**d**) PUU-III-10, (**e**) PUU-III-20.

For these composites, besides the aforementioned intelligent tests on performance, function, and shape memory, self-healing tests are also carried out. In this test, the self-healing effect of the sample is evaluated by tensile test. The stress–strain curves of PUU and other samples before and after self-repair are shown in Figure 8, Figure 9 and Figure 10. The self-repair efficiency (*R*_H_) is determined by tensile strength and elongation at break and defined as Equation (3). Additionally, the self-repair efficiency of three kinds of REOC composites is calculated by the equation (Table 1).

(3) $$R_{H}=\frac{\sigma_{H}\varepsilon_{H}}{\sigma_{0}\varepsilon_{0}}\times 100\%$$

*σ*_H_ and *σ*_0_ are the tensile strength of the repaired and initial samples, and *ε*_H_ and *ε*_0_ are the elongation at break of the repaired and initial samples.

Figure 8 shows the stress–strain curves of PUU and PUU-I composite materials before and after repairing at 130 °C. It can be seen that after heating the sample, the modulus is improved to a certain extent, which is owing to the fact that the sample is hardened from being soft by heating. According to Equation (3), the repair rate of PUU is 56.2%, while in PUU-I-2, the repair rate is greatly improved to about 94.3%. However, when an excessive amount is added, the repair rate decreases to some extent. It can be speculated that a small amount of REOC I can promote the movement of molecular chains, while an excessive amount affects the repair performance of PUU itself.

After REOC II is added, the self-healing rate of each sample is calculated by Equation (3). It is found that the self-healing effect of the polyurethane composite added with REOC II is better than that of the polyurethane composite added with REOC I.

Figure 9 shows the stress–strain curves of PUU and PUU-II composite materials before and after heating and repairing at 130 °C. As we can see, it is considered that the amino groups in the powder may promote the healing efficiency. Subsequently, the composite materials added with REOC III are tested, as shown in Figure 10. It is found that the self-healing efficiency of these composite materials is higher than that of the composite materials added with REOC II, since the urea group may further promote the compounding of the powder and polyurethane, which improves not only the mechanical properties but also the self-healing efficiency.

The self-healing of PUU is related to dynamic S–S bond exchange and hydrogen bond among urea groups. The existence of REOCs at a different dispersion status brings a different chain mobility and changes intermolecular forces. The increase in moduli of PUU composites might be caused by further reorganization of crystalline structures and dispersion status of REOCs during heating. Large aggregates of REOC I reduce the elongation at break of PUU-I composites. Although REOC II shows good miscibility with PUU, the distances of polymer chains are increased and intermolecular forces are reduced. Therefore, the tendency of S–S bond exchange and the reorganization of hydrogen bonds among urea groups are deteriorated. Finally, REOC III has mediate compatibility with PUU bonds, and its influence on self-healing mechanisms and mechanical properties is negligible.

## 4. Conclusions

REOCs with different ligands were used as fillers and mixed with polyurea–urethane (PUU) with disulfide bonds in main chains to create polymer composites having mechanical properties, luminescent functions, shape memory effect, and self-healing behaviors. The mechanisms of REOCs in the polymer matrix, which are highly determined by their ligands, greatly affected the performance of polymer composites. REOC I dispersed poorly in the PUU matrix and decreased the mechanical properties. The shape recovery ratio and self-healing efficiency were decreased as well. REOC II with amino groups is miscible with PUU, and the dispersion is improved. Mechanical properties are reduced, while intelligent behaviors are increased. REOC III having urea groups is partially miscible with PUU, and high loading content creates needling like morphologies. The mechanical properties can be maintained, while the shape memory effect is deteriorated. As far as luminescent functions are concerned, due to long organic ligands, REOC III and corresponding composites present low luminescence. This work is beneficial for the further design of suitable ligands for REOC to create polymer composites with all three features.

## Figures and Tables

**Table 1:** Tensile strength, elongation at break, fluorescence lifetime, shape recovery rates, and self-repair efficiency properties of samples.

Samples	Tensile Strength (MPa)	Elongation at Break (%)	Fluorescence Lifetime (s)	Shape Recovery Ratio (%)	Self-Healing Efficiency (%)
PUU	42	405	—	100	56.2
PUU-I-2	21	522	60	100	94.3
PUU-I-5	15	550	80	95	162.8
PUU-I-10	9	572	80	86	24.9
PUU-I-20	8	609	70	71	22.3
PUU-II-2	15	392	51	100	68.1
PUU-II-5	13	325	60	100	77.8
PUU-II-10	13	402	60	100	140.0
PUU-II-20	4	194	60	84	124.9
PUU-III-2	38	369	51	100	135.1
PUU-III-5	30	349	49	80	184.7
PUU-III-10	41	406	51	64	79.1
PUU-III-20	7	361	60	41	176.2

## Supplementary Materials

The following supporting information can be downloaded at: https://www.mdpi.com/article/10.3390/polym14102098/s1. Figure S1: FTIR spectra of REOC II and REOC III; Figure S2: HNMR curve of REOC II (DMSO); Figure S3: HNMR curve of REOC III (DMSO); Figure S4: Fluorescence lifetime and fitting curves of PUU-I composite materials: (a) PUU-I-2 (b) PUU-I-5 (c) PUU-I-10 (d) PUU-I-20; Figure S5: Fluorescence lifetime and fitting curves of PUU-II composite materials: (a) PUU-II-2 (b) PUU-II-5 (c) PUU-II-10 (d) PUU-II-20; Figure S6: Fluorescence lifetime and fitting curves of PUU-III composite materials: (a) PUU-III-2 (b) PUU-III-5 (c) PUU-III-10 (d) PUU-III-20.
