# Supplementary material for: Effects of Ligands in Rare Earth Complex on Properties, Functions, and Intelligent Behaviors of Polyurea–Urethane Composites
Source: Polymers (Basel). 2022 May 21;14(10):2098. doi: 10.3390/polym14102098 (PMC9143075; doi:10.3390/polym14102098)
Supplement: Supplementary file 1 [file polymers-14-02098-s001.zip › polymers-1709241-supplementary.pdf]

Supporting Information

Effects of Ligands in Rare Earth Complex on Properties, Functions, and Intelligent Behaviors of Polyurea–Urethane Composites

Lu Zhou [1,2,3], Hongwei Yang [1,2,3], Zhen Zhang [1,2,3], Yue Liu [4], Jayantha Epaarachchi [5], Zhenggang Fang [1,2,3,*], Liang Fang [1,2,3], Chunhua Lu [1,2,3] and Zhongzi Xu [1,2,3,*]

1 College of Materials Science and Engineering, Nanjing Tech University, Nanjing 211816, China; 201961103003@njtech.edu.cn (L.Z.); 201961203115@njtech.edu.cn (H.Y.); a773580419@163.com(Z.Z.); lfang@njtech.edu.cn (L.F.); chhlu@njtech.edu.cn (C.L.)

2 State Key Laboratory of Materials-Oriented Chemical Engineering, Nanjing Tech University, Nanjing 211816, China

3 Jiangsu Collaborative Innovation Center for Advanced Inorganic Function Composites, Nanjing Tech University, Nanjing 211816, China

4 Institute of Active Polymers, Helmholtz-Zentrum Hereon, Kantstr. 55, 14513 Teltow, Germany; yue.liu@hereon.de

5 School of Engineering, University of Southern Queensland, Toowoomba, QLD 4350, Australia; jayantha.epaarachchi@usq.edu.au

* Correspondence: zgfang@njtech.edu.cn (Z.F.); zzxu@njtech.edu.cn (Z.X.)

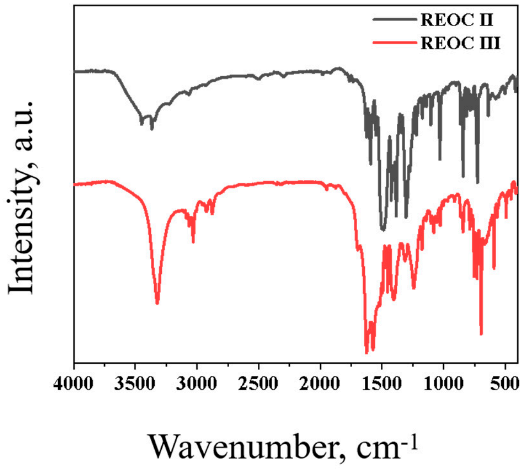

Figure S1. FTIR spectra of REOC II and REOC III.

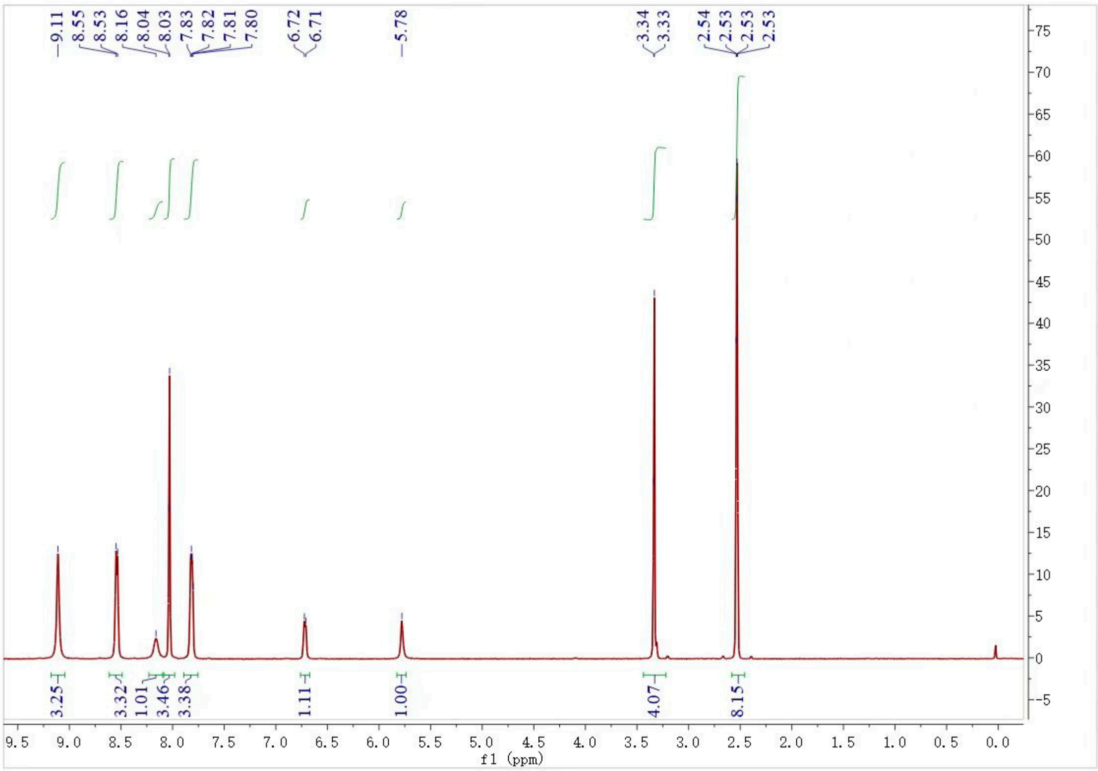

Figure S2. HNMR curve of REOC II (DMSO).

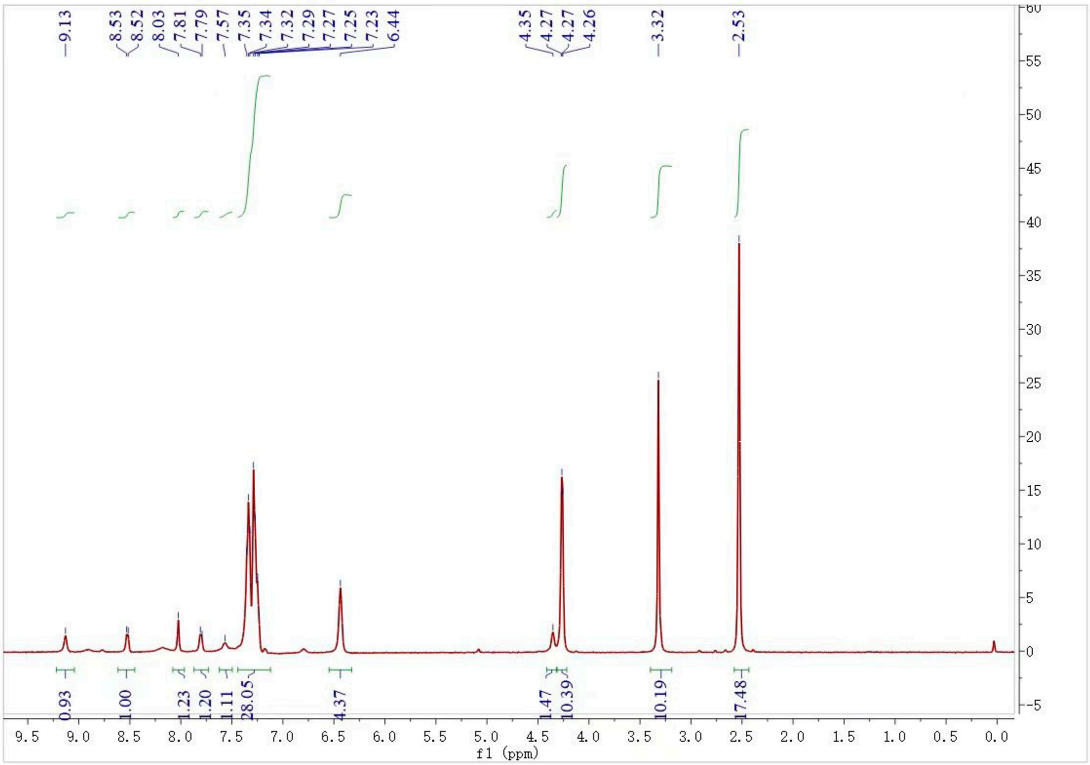

Figure S3. HNMR curve of REOC III (DMSO).

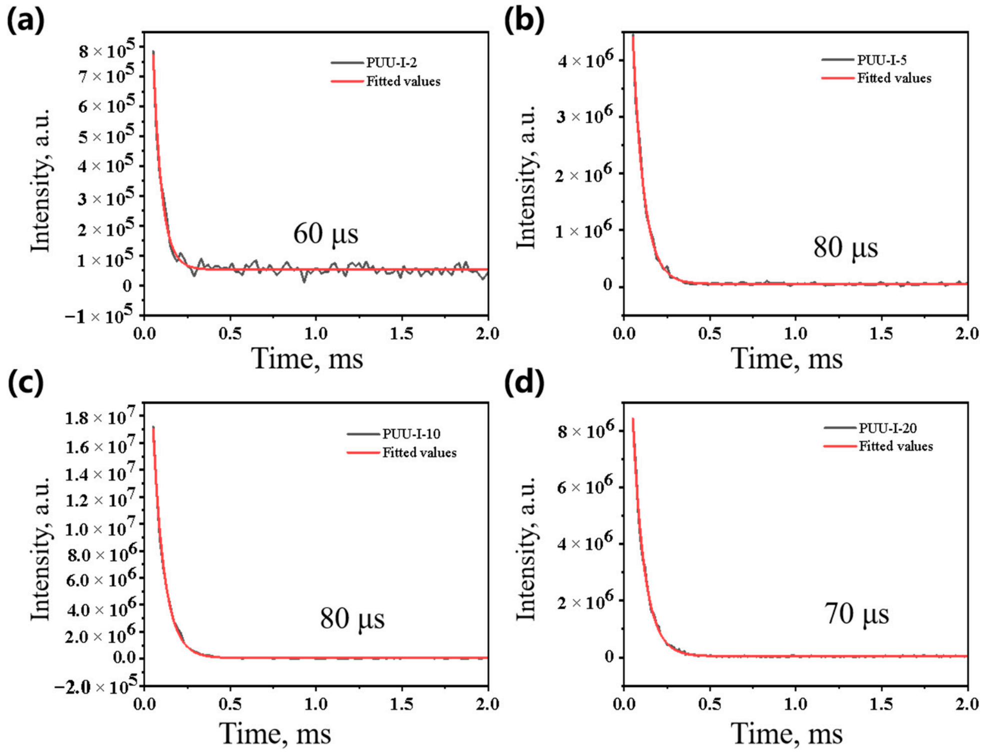

Figure S4. Fluorescence lifetime and fitting curves of PUU-I composite materials: (**a**) PUU-I-2 (**b**) PUU-I-5 (**c**) PUU-I-10 (**d**) PUU-I-20.

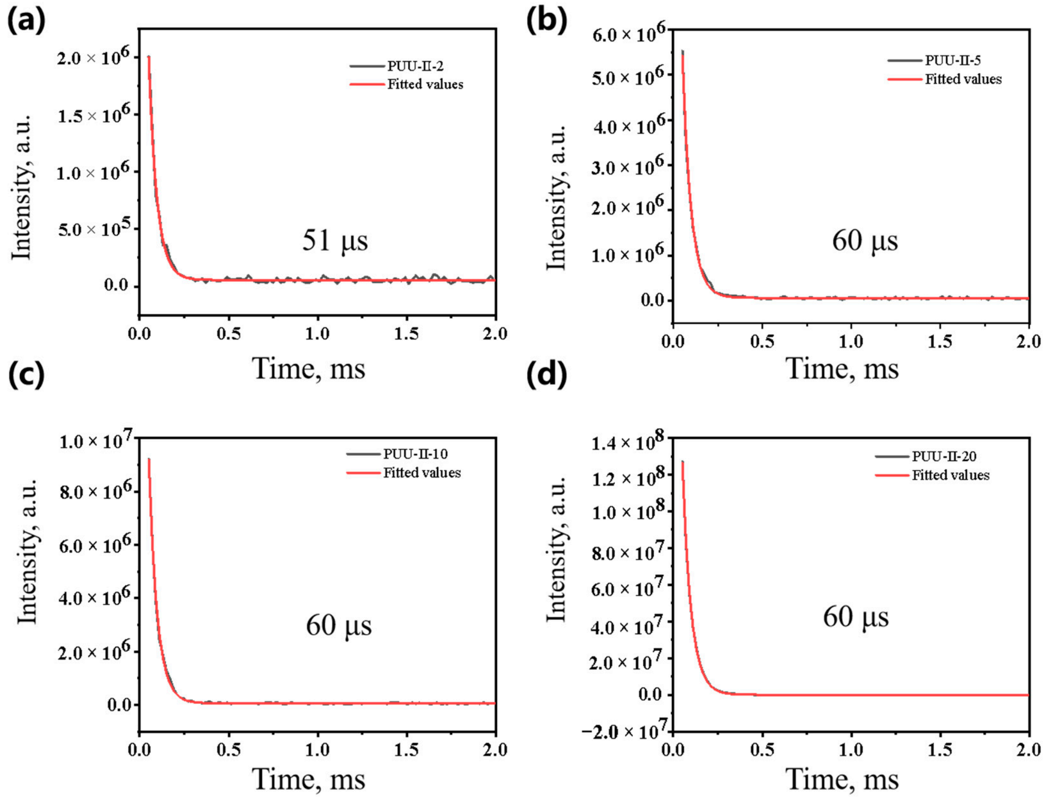

Figure S5. Fluorescence lifetime and fitting curves of PUU-II composite materials: (**a**) PUU-II-2 (**b**) PUU-II-5 (**c**) PUU-II-10 (**d**) PUU-II-20.

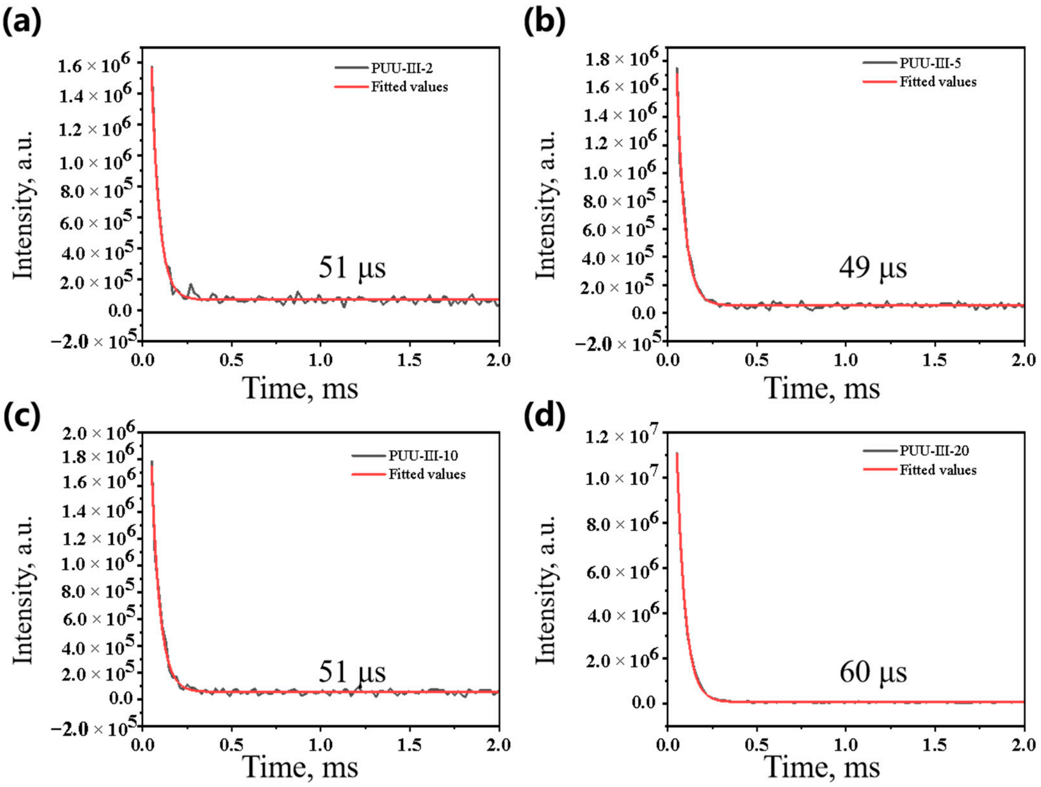

Figure S6. Fluorescence lifetime and fitting curves of PUU-III composite materials: (**a**) PUU-III-2 (**b**) PUU-III-5 (**c**) PUU-III-10 (**d**) PUU-III-20.
